# Drivers and Dynamics of Methicillin-Resistant Livestock-Associated Staphylococcus aureus CC398 in Pigs and Humans in Denmark

**DOI:** 10.1128/mBio.02142-18

**Published:** 2018-11-13

**Authors:** Raphael N. Sieber, Robert L. Skov, Jens Nielsen, Jana Schulz, Lance B. Price, Frank M. Aarestrup, Anders R. Larsen, Marc Stegger, Jesper Larsen

**Affiliations:** a Statens Serum Institut, Copenhagen, Denmark; b National Veterinary Institute, Technical University of Denmark, Kgs. Lyngby, Denmark; c Translational Genomics Research Institute, Flagstaff, Arizona, USA; d George Washington University, Washington, DC, USA; e National Food Institute, Technical University of Denmark, Kgs. Lyngby, Denmark; Emory University School of Medicine; Northern Arizona University

**Keywords:** CC398, MRSA, ST398, animal movements, livestock, molecular epidemiology, pigs, swine, zoonotic infections

## Abstract

The spread of livestock-associated methicillin-resistant Staphylococcus aureus clonal complex 398 (LA-MRSA CC398) within the Danish pig production system has been linked to an increased number of human infections. Yet, the population structure and transmission dynamics of this important pathogen remain poorly understood. In this study, whole-genome sequences from 371 LA-MRSA CC398 isolates collected between 2004 and 2015 were subjected to bioinformatic analyses. The isolates originated from Danish pig farms (*n* = 209) and people having livestock contact (*n* = 79). In addition, whole-genome sequence data from 82 isolates representing an international reference collection and 83 isolates from Danish patients were included in the analysis. The results demonstrated that the increasing prevalence of LA-MRSA CC398 in Danish pigs and patients was caused by clonal expansion of three dominant lineages. The results also showed that these lineages were enriched for the tetracycline resistance gene *tet*(K) and other determinants conferring resistance to some of the most frequently used antimicrobials in Danish pigs. The association between pig movements and the spread of LA-MRSA CC398 was assessed in a Poisson regression analysis of 17,009 pig movements into 273 farms with known LA-MRSA CC398 status. The results demonstrated that animal movements have played a critical role in the dissemination of LA-MRSA CC398 within the Danish pig production system, although other transmission routes may also have contributed. Consistent with this scenario, the genetic relatedness of isolates from different farms was positively correlated with the number of animal movements between the farms.

## Abstract (precis)

Livestock-associated methicillin-resistant Staphylococcus aureus clonal complex CC398 (LA-MRSA CC398) is resistant to nearly all β-lactams and several non-β-lactam antimicrobials. Over the last decade, it has become widespread in pig farms across Europe and is now an important cause of human infections in countries with previously low levels of MRSA, such as the Netherlands and Denmark. The hitherto uncontrolled spread of LA-MRSA CC398 underscores an urgent need to understand its epidemiology in order to develop evidence-based interventions. This study demonstrates that pig movements between farms in combination with increased bacterial resistance to specific antibiotics and heavy metals were important drivers of the rapid spread of LA-MRSA CC398 in the Danish pig production system. These findings should be taken into consideration when researchers and policy makers evaluate and decide on actions and policies to limit the spread of LA-MRSA CC398 and other pathogens in food animals.

## INTRODUCTION

Pigs are the primary host of livestock-associated methicillin-resistant Staphylococcus aureus CC398 (LA-MRSA CC398), which has been an increasing cause of human infections in Denmark and other European countries with industrial pig production (1–3). In humans, LA-MRSA CC398 is primarily associated with skin and soft tissue infections (SSTIs) but has also caused invasive disease, such as bloodstream infections (BSIs), and occasionally death (1–3). The Danish pig industry has a pyramidal structure in which nucleus and multiplier herds (here collectively referred to as breeding farms) at the apex produce and sell sows to production and weaner herds (here collectively referred to as production farms) at the bottom (4). In the first nationwide survey conducted in 2008 in Denmark, the prevalence of LA-MRSA CC398 was 0% and 3.5% in breeding and production farms, respectively, both of which increased to more than 60% by 2014 (5–7). In parallel, the number of human infections increased progressively, peaking in 2014, where LA-MRSA CC398 accounted for 16% and 21% of all MRSA BSIs and SSTIs, respectively (2, 3). The structure of the Danish pig production system and the national MRSA surveys of Danish pig farms are described in Text S1 in the supplemental material.

10.1128/mBio.02142-18.6 TEXT S1 Supplemental text and references. Download Text S1, DOCX file, 0.02 MB. Copyright © 2018 Sieber et al. 2018 Sieber et al. This content is distributed under the terms of the Creative Commons Attribution 4.0 International license.

The population structure and transmission dynamics of LA-MRSA CC398 within the Danish pig production system have not been well studied. Because its transmission pathways remain poorly understood, the causes of this epidemic are keenly debated, with various possible explanations for the observed farm-to-farm transmission, including movement of positive animals, inadequate control measures, and spread by humans, contaminated fomites, wind, insects, rodents, and other alternative hosts. Animal movements have long been considered a critical factor in the spread of livestock diseases (8, 9), and it has been suggested that animal movements may play a similar role in the dissemination of LA-MRSA CC398 (4, 10, 11).

The present study sought to understand the rapid spread of LA-MRSA CC398 in pigs and humans in Denmark. To accomplish this objective, whole-genome sequencing and analysis of pig movement data were used to infer the population structure and dynamics of LA-MRSA CC398 in the Danish pig production system and to identify genetic factors and epidemiological drivers associated with the success of this abundant human pathogen.

## RESULTS

### Prevalence and population dynamics of LA-MRSA CC398.

**FIG 1:**
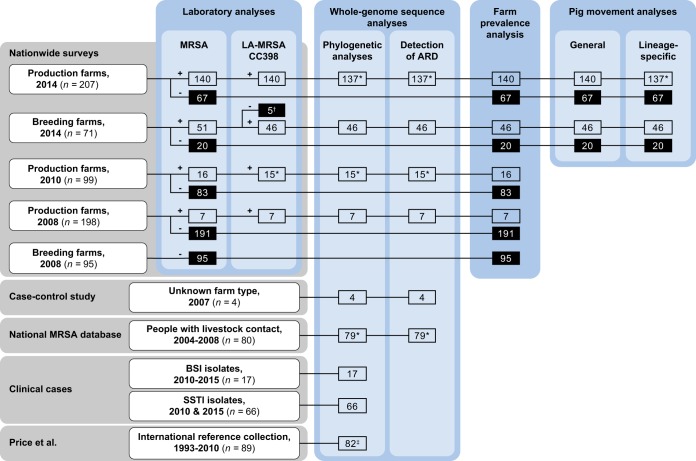
Schematic representation of the S. aureus CC398 collections (gray areas) and the different analyses (blue areas). Abbreviations: MRSA, methicillin-resistant Staphylococcus aureus; LA-MRSA, livestock-associated methicillin-resistant Staphylococcus aureus; CC, clonal complex; SSTI, skin and soft tissue infection; BSI, bloodstream infection; ARD, antimicrobial resistance determinants. *, three isolates from the 2014 survey, one isolate from the 2010 survey, and one isolate from people having livestock contact were unavailable for whole-genome sequencing and therefore excluded from these analyses. †, five farms from the 2014 survey were positive for MRSA CC1 and excluded from downstream analyses because their LA-MRSA CC398 status was unknown. ‡, five isolates were from people having livestock contact and two were from the 2008 survey.

**FIG 2:**
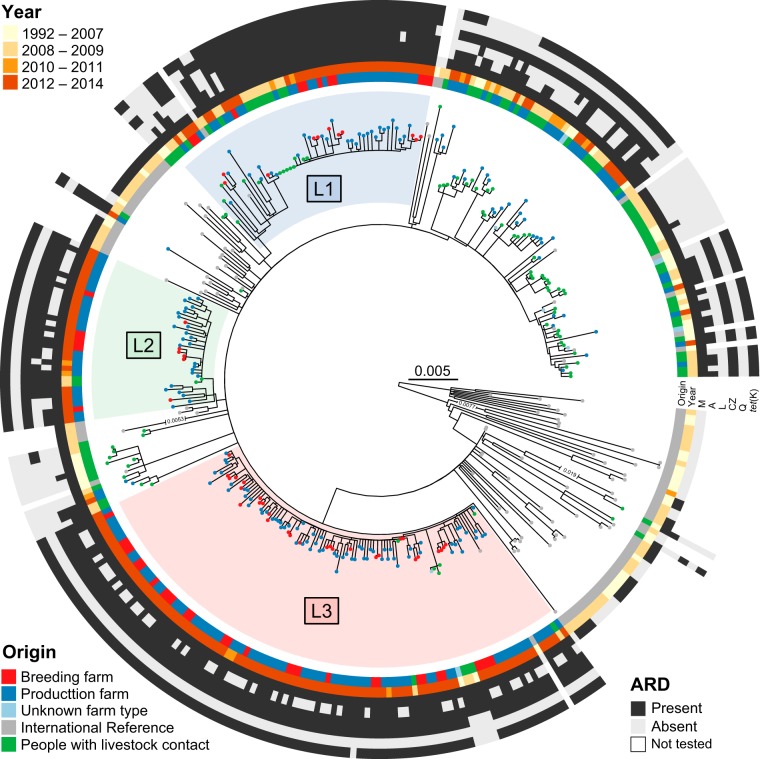
Maximum-likelihood phylogeny of 288 LA-MRSA CC398 isolates from this study and 82 S. aureus CC398 isolates from the international reference collection. The phylogeny was estimated for 5,364 variable sites after filtering for recombination tracts (824 SNPs), by using a GTR model of nucleotide substitution. Numbers in broken branches indicate the length by which the corresponding branch was reduced. The tree was rooted according to the work of Price et al. (13). The scale bar represents the number of nucleotide substitutions per variable site. Selected antimicrobial resistance phenotypes and determinants are shown. Abbreviations: LA-MRSA, livestock-associated methicillin-resistant Staphylococcus aureus; CC, clonal complex; SNP, single-nucleotide polymorphism; L1, lineage 1; L2, lineage 2; L3, lineage 3; ARD, antimicrobial resistance determinants; M, methicillin; A, aminoglycoside; L, lincosamide; CZ, cadmium/zinc; Q, quinolone.

MRSA was identified in 3.5% (7/198), 16.2% (16/99), and 67.6% (140/207) of the participating production farms in the 2008, 2010, and 2014 surveys, respectively (5–7). All isolates belonged to CC398 (5–7). In parallel, the proportion of breeding farms that tested positive for MRSA increased from 0.0% (0/95) in 2008 to 71.8% (51/71) in 2014 (5, 7). The vast majority (46/51) of isolates belonged to CC398, whereas the rest belonged to CC1 (7). The LA-MRSA CC398 status of the five breeding farms that tested positive for MRSA CC1 should be regarded as unknown because only one MRSA isolate per farm was subjected to genotypic characterization (see Text S1 in the supplemental material). Thus, LA-MRSA CC398 was identified in 69.7% (46/66) of the breeding farms with known LA-MRSA CC398 status in 2014. Of the 209 LA-MRSA CC398 isolates identified in these surveys, 205 were available for whole-genome sequencing (Fig. 1). In addition, four LA-MRSA CC398 isolates recovered from pig farms in 2007 (12) and 79 LA-MRSA CC398 isolates collected from people with livestock exposure between 2004 and 2008 (2) were subjected to whole-genome sequencing (Fig. 1). The phylogenetic relationship of the 288 study isolates described above as well as 82 S. aureus CC398 isolates from an international reference collection (13) is shown in Fig. 2, whereas Fig. S1 illustrates the phylogenetic relationship between the same isolates as in Fig. 1 and 83 LA-MRSA CC398 isolates from Danish patients who had an episode of either SSTI or BSI between 2010 and 2015 (3). The 288 study isolates are described in detail in Table S1.

10.1128/mBio.02142-18.1 FIG S1 Maximum-likelihood phylogeny of 288 LA-MRSA CC398 isolates from this study, 82 S. aureus CC398 isolates from the international reference collection, and 83 LA-MRSA CC398 isolates from Danish SSTI and BSI patients. The phylogeny was estimated for 5,529 variable sites after filtering for recombination tracts (556 SNPs), by using a GTR model of nucleotide substitution. Numbers in broken branches indicate the length by which the corresponding branch was reduced. The tree was rooted according to the work of Price et al. (13). Bootstrap values above 90% are illustrated by filled circles at the nodes. The scale bar represents the number of nucleotide substitutions per variable site. Abbreviations: LA-MRSA, livestock-associated methicillin-resistant Staphylococcus aureus; CC, clonal complex; SSTI, skin and soft tissue infection; BSI, bloodstream infection; SNP, single-nucleotide polymorphism; L1, lineage 1; L2, lineage 2; L3, lineage 3; AT, Austria; BE, Belgium; CA, Canada; CH, Switzerland; CN, China; DE, Germany; DK, Denmark; ES, Spain; FI, Finland; FR, France; GF, French Guiana; HU, Hungary; IT, Italy; NL, the Netherlands; PE, Peru; PL, Poland; PT, Portugal; SI, Slovenia; US, United States. Download FIG S1, PDF file, 0.6 MB. Copyright © 2018 Sieber et al. 2018 Sieber et al. This content is distributed under the terms of the Creative Commons Attribution 4.0 International license.

10.1128/mBio.02142-18.3 TABLE S1 Description of 288 LA-MRSA CC398 isolates from this study. Download Table S1, XLS file, 0.2 MB. Copyright © 2018 Sieber et al. 2018 Sieber et al. This content is distributed under the terms of the Creative Commons Attribution 4.0 International license.

**FIG 3:**
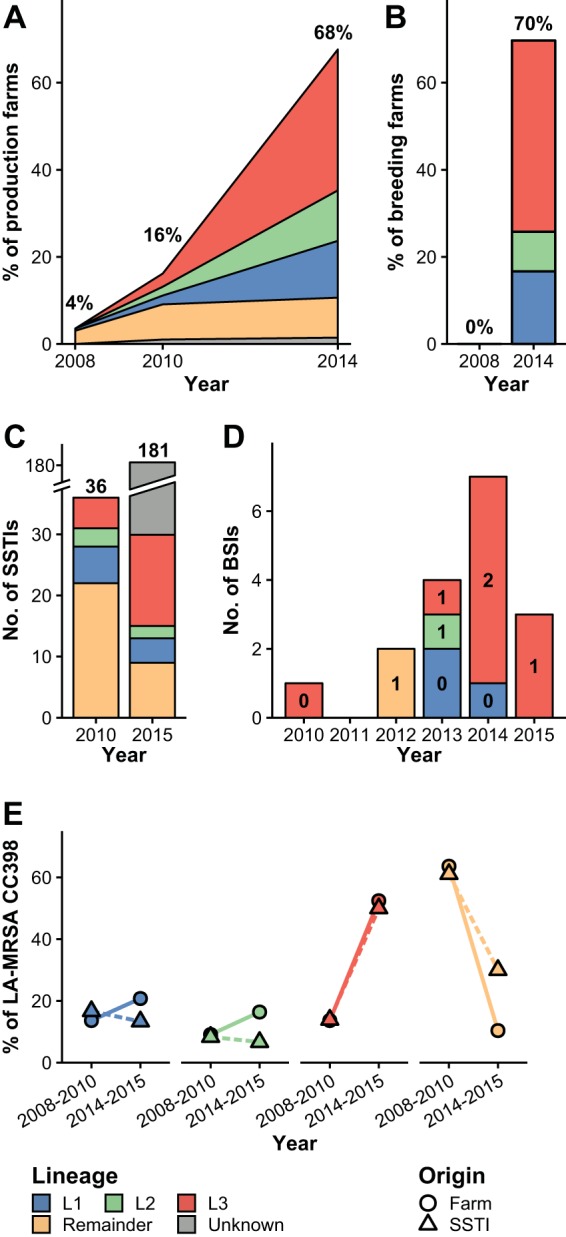
Temporal trends of LA-MRSA CC398 in Danish pig farms and patients, 2008 to 2015. (A) Prevalence of LA-MRSA CC398 in production farms. LA-MRSA CC398 isolates from four farms were not available for whole-genome sequencing, and the lineage was therefore regarded as unknown. (B) Prevalence of LA-MRSA CC398 in breeding farms with known LA-MRSA CC398 status. (C) Number of LA-MRSA CC398 SSTIs caused by L1 to L3 or other isolates among all 36 cases registered in 2010 and a subset of 30 out of 181 cases registered in 2015. (D) Number of LA-MRSA CC398 BSIs caused by L1 to L3 or other isolates among all 17 cases registered between 2010 and 2015. Numbers in bars indicate deaths attributable to LA-MRSA CC398 BSI. (E) Proportion of L1, L2, L3, and other isolates among whole-genome-sequenced LA-MRSA CC398 isolates originating from the pig farm surveys conducted in 2008, 2010, and 2014 and SSTI cases registered in 2010 and 2015. Abbreviations: LA-MRSA, livestock-associated methicillin-resistant Staphylococcus aureus; CC, clonal complex; SSTI, skin and soft tissue infection; BSI, bloodstream infection; L1, lineage 1; L2, lineage 2; L3, lineage 3.

In 2014, 86.1% of the available LA-MRSA CC398 isolates from production farms clustered into three dominant lineages (here termed L1, L2, and L3) (Fig. 2 and Fig. S1). L1 to L3 were found in 13.0% (27/207), 11.6% (24/207), and 32.4% (67/207) of the production farms tested in 2014, respectively, whereas isolates from 9.2% (19/207) of the farms clustered outside L1 to L3 (Fig. 3A). Although L1 to L3 were identified as early as in 2008, 2008, and 2006, respectively, they constituted only 28.4% (31/109) of the isolates collected from pigs and humans between 2004 and 2010 (Fig. 2 and Fig. S1). For breeding farms, all 46 available LA-MRSA CC398 isolates from the 2014 survey clustered into L1 to L3, with L1, L2, and L3 being present in 16.7% (11/66), 9.1% (6/66), and 43.9% (29/66) of the farms, respectively (Fig. 3B). L3 underwent the most rapid spread in Danish production farms during 2010 to 2014 and had become the most abundant lineage in both production and breeding farms by 2014 (Fig. 3A and B). By chance, 31 farms (12 production farms and 19 breeding farms) were included in more than one of the three surveys. However, none of the farms tested positive more than once (Table S2).

10.1128/mBio.02142-18.4 TABLE S2 List of pig farms that participated in ≥1 survey. Download Table S2, DOCX file, 0.02 MB. Copyright © 2018 Sieber et al. 2018 Sieber et al. This content is distributed under the terms of the Creative Commons Attribution 4.0 International license.

L1 to L3 were all well supported with bootstrap values >90% (Fig. S1). L1 and L3 were predominantly associated with *spa* type t034, and each contained a methicillin-susceptible S. aureus (MSSA) CC398 isolate from Denmark, both of which belonged to the international reference collection (Fig. S1). In contrast, L2 consisted entirely of LA-MRSA CC398 isolates from this study, most of which had *spa* type t011.

### Associations between pig movements and presence of LA-MRSA CC398.

In Denmark, it has been mandatory to record all pig movements in the Central Husbandry Register (CHR) since January 2002 (14). All 2.76 million pig movements registered between 2011 and 2014 were reviewed and analyzed. The analysis showed that an average breeding farm delivered animals to 4.7 times more production farms per year than an average production farm (5.90 versus 1.25 production farms per year, respectively; *P < *0.0001).

**FIG 4:**
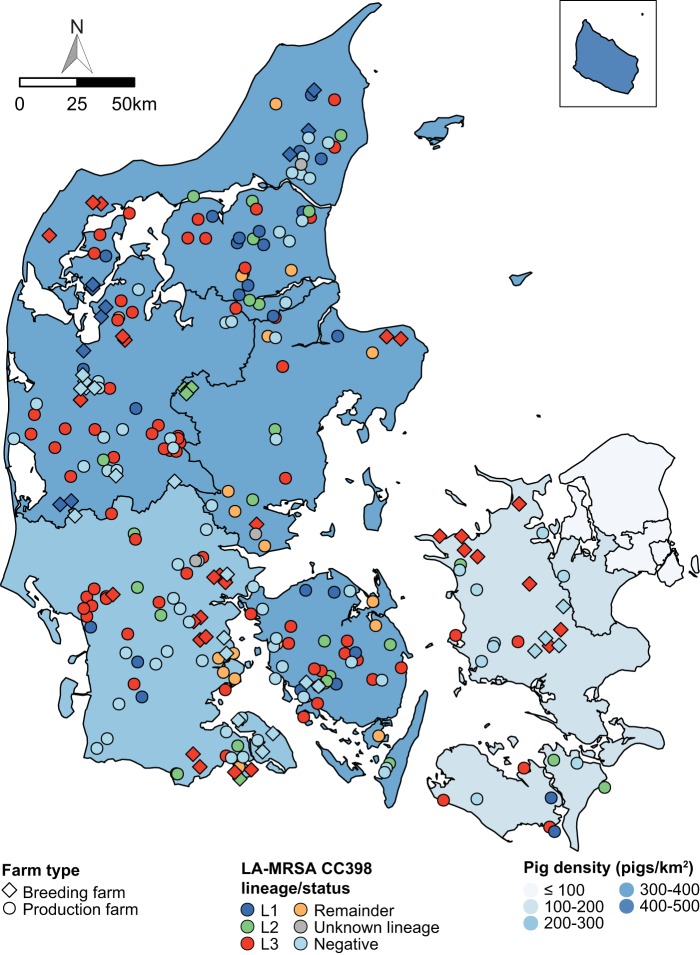
Spatial distribution of pig farms from the 2014 survey. Each farm was placed randomly within a 5-km radius of the exact CHR address to protect anonymity. The pig density per km^2^ is shown for each province. Abbreviations: LA-MRSA, livestock-associated methicillin-resistant Staphylococcus aureus; CC, clonal complex; L1, lineage 1; L2, lineage 2; L3, lineage 3. The administrative boundaries are from EuroGeographics.

**FIG 5:**
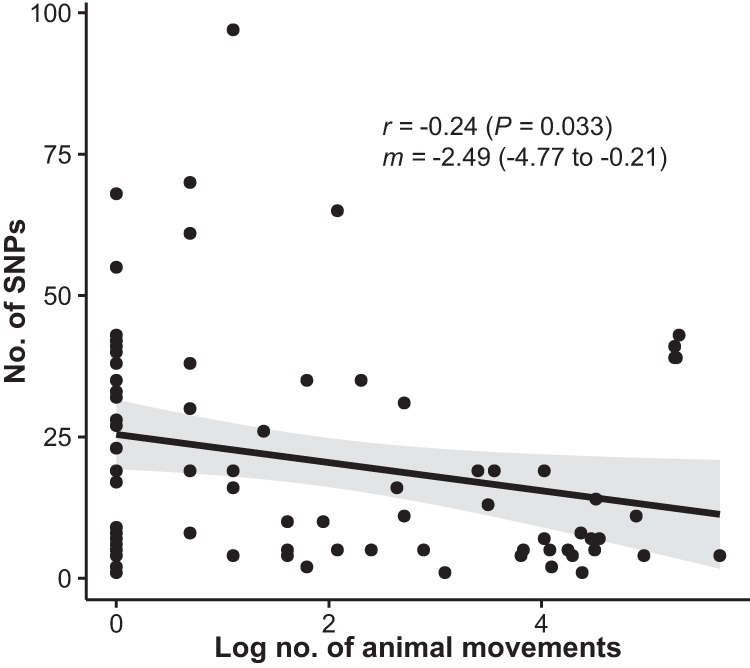
Relationship of the natural logarithmic number of animal movements between dyads of supplying and receiving farms that participated in the 2014 survey and the number of SNPs between the corresponding LA-MRSA CC398 isolate pairs. Pearson’s correlation coefficient (*r*) with *P* value in parentheses, linear regression line (solid) with 95% CIs (gray area), and slope (*m*) with 95% CIs are shown. Abbreviations: LA-MRSA, livestock-associated methicillin-resistant Staphylococcus aureus; CC, clonal complex; SNP, single-nucleotide polymorphism; 95% CI, 95% confidence interval.

An overdispersed Poisson regression model was used to analyze the association between pig movements occurring during 2011 to 2014 and the incidence of LA-MRSA CC398 on pig farms that participated in the 2014 survey. The data set consisted of 17,009 pig movements into 273 farms, including 190 of the 207 production farms and 53 of the 66 breeding farms. The five breeding farms that tested positive for MRSA CC1 were excluded from the analysis because their LA-MRSA CC398 status was unknown (Fig. 1). The locations of the 273 farms and their LA-MRSA CC398 status are illustrated in Fig. 4, whereas Fig. S2 shows pig movements into the same farms. The analysis revealed that farms receiving animals from positive farms had a >4-fold-higher incidence rate than farms receiving animals from negative farms (incidence rate ratio, 4.22; 95% confidence interval [CI], 3.29 to 5.41; *P < *0.0001) (Table 1). The background incidence rate for farms that did not receive animals from other farms was estimated to be 0.23 (95% CI, 0.18 to 0.29). Poisson regression analysis further showed that farms receiving animals from farms that tested positive for L1 and L3 had significantly higher incidence rates for those lineages than farms receiving animals from negative farms (Table 1). In contrast, farms receiving animals from farms that were positive for L2 did not have a higher incidence rate than farms receiving animals from negative farms (Table 1). These results suggest that pig movements played a crucial role in the spread of LA-MRSA CC398 between farms. In accord with this view, the genetic distance (i.e., the number of single-nucleotide polymorphisms [SNPs]) between LA-MRSA CC398 isolate pairs from a supplying and receiving farm was negatively correlated with the number of movements between the farms (Pearson’s correlation coefficient, −0.24; *P = *0.033) (Fig. 5).

**TABLE 1:** Incidence rate ratios of LA-MRSA CC398^*b*^

LA-MRSA CC398 status (receiving farm)	LA-MRSA CC398 status (supplying farm)	IRR	95% CI	*P* value^*a*^
Positive	Negative	Reference		
	Positive	4.22	3.29–5.41	<0.0001
Positive for L1	Negative	Reference		
	Positive for L1	9.88	4.66–20.9	<0.0001
	Positive for other than L1	0.026	0.003–0.201	0.0005
Positive for L2	Negative	Reference		
	Positive for L2	14.9	0–∞	
	Positive for other than L2	5.24	0–∞	
Positive for L3	Negative	Reference		
	Positive for L3	2.39	1.40–4.09	0.0015
	Positive for other than L3	0.277	0.151–0.506	<0.0001

a Only *P* values < 0.05 are shown.

b Abbreviations: L1, lineage 1; L2, lineage 2; L3, lineage 3; IRR, incidence rate ratio; 95% CI, 95% confidence interval.

10.1128/mBio.02142-18.2 FIG S2 Animal movements into pig farms from the 2014 survey. The network represents 17,009 pig movements into 273 farms with known LA-MRSA CC398 status, including 190 of the 207 production farms and 53 of the 66 breeding farms, over a 4-year period from 2011 to 2014. Arrows indicate the direction of pig movements. Abbreviations: LA-MRSA, livestock-associated methicillin-resistant Staphylococcus aureus; CC, clonal complex; L1, lineage 1; L2, lineage 2; L3, lineage 3. Download FIG S2, PDF file, 0.6 MB. Copyright © 2018 Sieber et al. 2018 Sieber et al. This content is distributed under the terms of the Creative Commons Attribution 4.0 International license.

### Presence of antimicrobial resistance determinants.

The genetic foundation for the successful spread of the three dominant lineages was investigated by comparing the prevalence of antimicrobial resistance determinants in L1 to L3 isolates combined versus the remainder. The analysis revealed that L1 to L3 were enriched for determinants conferring resistance to aminoglycosides, lincosamides, cadmium/zinc, and quinolones compared to the remainder (Fig. S1 and Table S3). In addition, L1 to L3 isolates were enriched for the tetracycline resistance gene *tet*(K), whereas another tetracycline resistance gene, *tet*(M), was ubiquitous in both L1 to L3 and the remainder. The *lnu*(B) and *czrC* genes conferring resistance to lincosamides and cadmium/zinc, respectively, were evenly distributed in L1 to L3. The *gyrA* mutations conferring resistance to quinolones were present only in L1. Aminoglycoside resistance was mainly encoded by *aadD* and *aadE* in L1, whereas *str* was the most abundant aminoglycoside resistance gene in L2 and L3 (Fig. S1 and Table S3).

10.1128/mBio.02142-18.5 TABLE S3 Prevalence of antimicrobial resistance determinants in the dominant lineages (L1 to L3) and remainder. Download Table S3, DOCX file, 0.02 MB. Copyright © 2018 Sieber et al. 2018 Sieber et al. This content is distributed under the terms of the Creative Commons Attribution 4.0 International license.

### Population structure and dynamics of LA-MRSA CC398 in humans.

The temporal distribution of L1 to L3 was investigated in a Danish collection of 83 LA-MRSA CC398 isolates from patients who had an episode of SSTI or BSI, including all 17 BSI cases registered between 2010 and 2015, all 36 SSTI cases registered in 2010, and 30 of 181 SSTI cases registered in 2015 (3). The maximum-likelihood phylogeny (Fig. S1) showed that the proportion of SSTIs caused by L1 to L3 isolates increased from 38.9% (14/36) in 2010 to 70.0% (21/30) in 2015 (Fig. 3C). In addition, L1 to L3 accounted for 88.2% (15/17) LA-MRSA CC398 BSIs during 2010 to 2015, including five of six cases with fatal outcomes (Fig. 3D). L3 was associated with the largest increase in the number of SSTIs and for 10 of 17 BSIs during 2010 to 2015 (Fig. 3C and D). Of note, the relative abundance of L3 isolates in the Danish pig production system and among SSTI cases increased at the same rate (Fig. 3E).

## DISCUSSION

Based on simulation models and network analyses, previous work has shown that pig movements and herd types are likely to play important roles in the spread of swine diseases in Denmark (4, 8). In addition, several small-scale studies from Denmark and the Netherlands have found that the LA-MRSA CC398 status of a given pig farm depends largely on the LA-MRSA CC398 status of its suppliers (10, 15, 16). This study shows on a national scale that animal movements were indeed associated with the rapid spread of LA-MRSA CC398 within the Danish pig production system and that farms receiving animals from negative farms had a significantly reduced risk for becoming positive for LA-MRSA CC398.

Most of the LA-MRSA CC398 isolates from the 2014 survey belonged to the three dominant lineages (L1 to L3), whereas isolates from previous years were more heterogeneous. This suggests that the rapid spread of LA-MRSA CC398 within the Danish pig production system was caused by clonal expansion of the three lineages. Breeding herds are considered a critical factor in the spread of swine diseases because they produce and supply hybrid breeding pigs to a large number of production holdings (4). Thus, it is possible that the emergence of the same lineages in breeding farms somewhere between 2008 and 2014 has accelerated the spread of LA-MRSA CC398 within the Danish pig production system.

L1 and L3 each contained an MSSA CC398 isolate from Denmark. The L1 isolate was collected from a Danish pig farm in 2008, whereas the L3 isolate originated from a Danish pig farm in 2002, 2 years before LA-MRSA CC398 was first recognized in Denmark (2). These observations could be due to spontaneous loss of *mecA*, which is known to occur both *in vivo* and during storage (17–19). However, it is also possible that the two isolates were part of an ancestral MSSA CC398 population (20).

Whereas L1 and L3 seem to spread predominantly by animal movements, our results indicate that animal movements played a less significant role in the spread of L2. In addition, animal movements cannot explain how LA-MRSA CC398 was introduced into breeding farms, as they do not receive animals from production farms. These observations support the idea that LA-MRSA CC398 can spread by other means of transmission, for example, through human carriers and contaminated fomites (11) or via the environment (21).

L1 to L3 were enriched for antimicrobial resistance determinants, including *czrC* and *tet*(K), both of which are integrated into the J1 region of the type Vc (5C2&5) staphylococcal cassette chromosome *mec* (SCC*mec*) element (22), meaning that they are genetically linked to the *mecA* gene conferring resistance to β-lactam antibiotics. In the Danish pig production system, zinc is commonly used as a feed additive at low doses and used at high doses for treatment of diarrhea during weaning, whereas tetracycline is the most frequently used antibiotic (23). Thus, the clonal expansion of L1 to L3 was likely driven by the heavy use of these antimicrobial agents, a view corroborated by the findings that the presence of *czrC* and the copresence of *tet*(K) and *tet*(M) confer a fitness advantage to LA-MRSA CC398 during exposure to zinc and tetracycline, respectively (24, 25). Of note, resistance to several antimicrobial classes, including aminoglycosides, tetracycline, and macrolides, was conferred by different genes in the three predominant lineages, indicating that LA-MRSA CC398 has followed different evolutionary courses to adapt to antimicrobial exposure. This process of parallel evolution further highlights the importance of selection by antimicrobial compounds used in the farm environment.

The clonal expansion of L1 to L3 in the Danish pig production system between 2010 and 2014 was reflected by a parallel increase in the number of human infections caused by these lineages. The introduction of LA-MRSA CC398 into the community, as documented previously (2, 3), is an expected outcome of a rapidly expanding pig reservoir. The public health and economic consequences of LA-MRSA CC398 are most pronounced in countries with low baseline levels of MRSA, such as Denmark and the Netherlands. To prevent transmission of LA-MRSA CC398 into hospitals and other health care facilities, the Danish Health Authority released updated guidelines for the management of MRSA in 2012 (26). These updated guidelines recommend targeted screening of pig farmers and their household members at the time of admission to the hospital. Subsequently, the Danish Institute for Local and Regional Government Research showed that the estimated health care costs incurred by LA-MRSA CC398 were DKK 43 million ($7 million) in 2014, corresponding to DKK 20,000 ($3,000) per patient (27).

The present study has limitations that need to be considered. The available MRSA collections from the three national surveys of pig farms consisted of a single MRSA isolate from a single time point from each positive farm. In addition, only a small proportion of the Danish pig farms was included in each survey. By discounting the possible copresence of genetically diverse MRSA isolates on the same farm, the true prevalence of LA-MRSA CC398 and the different lineages may have been underestimated. For example, it cannot be ruled out that LA-MRSA CC398 was present in the five breeding farms that tested positive for MRSA CC1 or that multiple LA-MRSA CC398 lineages were present on positive farms. Another limitation was that the small number of animal isolates prior to 2008 has hampered our ability to estimate the rise of the different LA-MRSA CC398 lineages in the Danish pig production system. To account for this, 79 isolates collected from people with livestock exposure between 2004 and 2008 were included in the analysis. It is possible, however, that some of these persons have acquired LA-MRSA CC398 from other sources than Danish pigs.

In summary, pig movements facilitated the spread of a few LA-MRSA CC398 lineages that were highly adapted to the selection pressures exerted by antimicrobial use in pigs, although other transmission routes (e.g., humans, contaminated fomites, and the environment) could have played minor roles. In light of the pyramidal structure of the Danish pig production system and the epidemiologic history of LA-MRSA CC398, it seems reasonable to argue that the introduction of these dominant lineages into breeding farms has accelerated their spread in the pig reservoir and from there to humans.

## MATERIALS AND METHODS

### Study isolates.

The isolates used in this study were obtained from different collections and studies (Fig. 1). The Danish LA-MRSA CC398 collection included pig farm isolates (one per farm) originating from three nationwide surveys conducted in 2008 (5), 2010 (6), and 2014 (7), respectively. Four pig farm isolates from a case-control study conducted in 2007 (12) and 79 isolates from people who were registered in the national MRSA database as having had occupational contact with livestock and having been colonized or infected with LA-MRSA CC398 between 1 January 2004 and 31 December 2008 were included to cover the period between the emergence of LA-MRSA CC398 in 2004 and the first nationwide pig farm survey conducted in 2008 (2). Finally, for comparative purposes, whole-genome sequence data from 82 isolates from an international reference collection (13) and 83 isolates from Danish patients who had an episode of either SSTI or BSI between 2010 and 2015 (3) were downloaded from the NCBI Sequence Read Archive (SRA) via BioProject accession number PRJNA274898 and PRJEB19505, respectively.

### Whole-genome sequencing and bioinformatic analyses.

A total of 283 LA-MRSA CC398 isolates were sequenced on different Illumina platforms as described previously (3). Mapping of sequence reads and SNP calling against the ST398 reference genome (strain SO385; GenBank accession no. AM990992) were carried out using NASP (28). To avoid confounding of phylogenetic interpretations, SNPs falling into regions of putative recombination, such as the ∼123-kb region that was horizontally acquired from a CC9 donor (13) or where ≥3 unique consecutive SNPs occurred in a single or few isolates, were removed from the alignment. R package phangorn version 2.4.0 (29) was used to estimate maximum-likelihood phylogenetic trees from the remaining sites using the best-fit model of nucleotide substitution under the Akaike and Bayesian information criteria. The resulting GTR substitution model was used together with stochastic tree rearrangement during the optimization processes. The robustness of the phylogenies was assessed with bootstrap analysis using 1,000 replicates, and the trees were rooted according to the work of Price et al. (13).

The presence of antimicrobial resistance determinants was investigated with Mykrobe predictor version 0.4.3 (30). Antimicrobial resistance genes were identified by comparing the whole-genome de Bruijn graph from each of the sequenced isolates with reference graphs generated from the ResFinder database (accessed 17 September 2017) (31) and the *czrC* gene encoding resistance to cadmium and zinc (GenBank accession no. KF593809). Genes classified as present by Mykrobe predictor were further filtered for coverage (≥80%) and median depth (≥5×). Point mutations were identified using default settings (accessed 17 September 2017).

### Collection of pig movement data.

Pig movement data as well as relevant information for each farm (type, location, number of animal movements, number of moved animals, and farm contacts) were extracted from CHR (14). For the Poisson regression analysis, the data set was restricted to pig movements that met the following criteria: (i) the receiving farm was tested for LA-MRSA CC398 in the 2014 survey (Fig. 1), (ii) the movement took place between 1 January 2011 and the sampling date of the recipient farm, and (iii) the supplying and receiving farms were registered as a nucleus, multiplier, production, or weaner herd. The final data set consisted of 17,009 movements into 243 farms, including 53 breeding farms and 190 production farms, and contained information on the test result of the supplying and receiving farm in the 2014 survey, the number of moved animals, and the time span between the date when the animals were moved and the sampling date of the receiving farm.

### Mapping of farms.

The spatial distribution of pig farms that participated in the 2014 survey was analyzed with R version 3.5.0 (32) by plotting their georeferenced CHR addresses as point data on digital maps along with the pig density per km^2^ for each province. Each data point was placed randomly within a 5-km radius of the exact CHR address to protect anonymity of the farm. Geodata (https://ec.europa.eu/eurostat/web/gisco/geodata/reference-data/administrative-units-statistical-units/nuts#nuts03) were downloaded from EuroGeographics, whereas data on the number of pigs (http://www.statbank.dk/HDYR07) and area (https://www.statbank.dk/ARE207) for each province were obtained from Statistics Denmark.

### Statistical analyses.

Statistical analyses were performed with R version 3.5.0 (32). All reported statistical tests were 2-sided, and *P* values < 0.05 were considered statistically significant. Pearson’s correlation coefficient was used to evaluate the relationship of the logarithmic number of animal movements between dyads of supplying and receiving farms and the number of SNPs between the corresponding LA-MRSA CC398 isolate pairs. Variables were compared between groups using the Student *t* test for continuous data and Fisher’s exact test for categorical data. An overdispersed Poisson regression model was used to analyze the effect of the LA-MRSA CC398 status of the supplying farm on the LA-MRSA CC398 status of the receiving farm. The model was fitted with the two covariates, number of moved animals and the time span between the date when the animals were moved and the sampling date of the receiving farm, as well as all possible interaction terms. Three movements were excluded as outliers after preliminary analysis, due to excessive weight caused by exceptionally high numbers of moved animals. The results are presented as incidence rate ratios with 95% CIs.

### Data availability.

The whole-genome sequence data generated in this study have been submitted to the European Nucleotide Archive under BioProject accession number PRJEB25608. Data sets and R scripts for all analyses and figures are available at SourceForge (https://sourceforge.net/p/la-mrsa-cc398-in-pigs-humans).
