# Supplementary material for: Drivers and Dynamics of Methicillin-Resistant Livestock-Associated Staphylococcus aureus CC398 in Pigs and Humans in Denmark
Source: mBio. 2018 Nov 13;9(6):e02142-18. doi: 10.1128/mBio.02142-18 (PMC6234867; doi:10.1128/mBio.02142-18)
Supplement: TEXT S1 [file mbo005184157s1.docx]

# SUPPLEMENTAL TEXT

# SUPPLEMENTAL TEXT 1: STRUCTURE OF THE DANISH PIG PRODUCTION SYSTEM

Danish production holdings can be divided into three categories: integrated holdings with both sows and finishers (i.e., farrow-to-finish); sow holdings (i.e., farrow-to-weaner holdings); and finisher holdings without sows (i.e., weaner-to-finish, and finisher holdings). In 2014, there were 3,638 production holdings in Denmark, comprising 6,623 individual herds: 1,122 integrated holdings (average, 492 sows and 6,026 finishers per holding); 684 sow holdings (average, 701 sows 714 finishers per holding); and 1,832 finisher holdings (average, 0 sows and 6,529 finishers) (1, 2). In total, Denmark produced ≈18 million slaughter pigs and exported ≈10 million weaners in 2014 (13).

Danish breeding holdings comprise both nucleus and multiplier herds. Nucleus herds generate genetic improvement and deliver future purebred breeding pigs to multiplier herds, which produce and deliver hybrid breeding pigs to production holdings. There were 260 breeding herds in Denmark in 2014, including 71 nucleus herds and 189 multiplier herds (3).

# SUPPLEMENTAL TEXT 2: NATIONAL MRSA SURVEYS OF DANISH PIG FARMS

The Danish Veterinary and Food Administration performed MRSA surveys in pig farms in 2008 on request from the European Commission (question no. EFSA-Q-2008-417A) and in 2010 and 2014 as part of a national MRSA surveillance program (4-6, 7).

The objective of the 2008 survey was to estimate the prevalence of MRSA in breeding holdings and production holdings with ≥50 breeding pigs within the European Union. The sample sizes were calculated based on the total number of holdings with breeding pigs in each Member State, an expected prevalence of 50%, a desired confidence level of 95%, and a desired accuracy of 7.5%. In Denmark, 198 breeding holdings (i.e., nucleus and multiplier holdings) and 3,457 production holdings (i.e., farrow-to-weaner and farrow-to-finish holdings) met the eligibility criteria. Of the eligible holdings, 95 (48%) breeding holdings and 198 (6%) production holdings were randomly selected to ensure that the survey included holdings from all regions where pigs are raised. Within each holding, environmental dust samples were taken from the immediate environment of five individual pens with breeding pigs. The five dust swabs were pooled in 100 ml of Mueller-Hinton broth supplemented with 6.5% NaCl and incubated at 37°C for 16-20 h. One milliliter of pre-enrichment culture was then inoculated into 9 ml of tryptic soy broth containing 3.5 mg/l cefoxitin and 75 mg aztreonam and incubated for a further 16-20 h at 37°C. A 10-µl loop-full of selective enrichment culture was then spread onto a chromogenic agar selective for MRSA and incubated for 24-48 h at 37°C. Based on colony morphology and color, up to five presumptive MRSA isolates were sub-cultivated on blood agar. One presumptive *S. aureus* isolate was subjected to confirmatory testing for *S. aureus* and MRSA using a multiplex PCR assay or two independent PCR assays for detection of a *S. aureus*-specific gene and the *mecA* gene. If the first isolate of the initial five isolates was not identified as MRSA, the next isolate was tested until MRSA was identified or all five isolates had been tested.

The 2010 survey investigated the prevalence of MRSA in production herds with ≥50 slaughter pigs, whereas the aim of the 2014 survey was to estimate the prevalence of MRSA in breeding herds and production herds with ≥50 slaughter pigs. Sample size calculations were not performed. Production herds were selected from lists of eligible production herds by the local Veterinary Inspection Units to ensure that the herds represented all regions where pigs are raised. There were no further requirements for selecting the herds, and the production herds should therefore be regarded as convenience samples. The samples represented 1.2% (99/7,940) and 3.1% (207/6,623) of the total number of production herds in Denmark in 2010 and 2014, respectively. The sample of breeding herds in the 2014 survey included all nucleus herds (*n*=71), whereas multiplier herds were not investigated. Within each herd, nasal swabs were taken from the anterior nares of five individual slaughter pigs in each of five individual pens. The five nasal swabs from each pen were pooled and analyzed for MRSA in the same way as in the 2008 survey. If the first pool was negative for MRSA, the next pool was tested until MRSA was identified or all five pools had been tested.

**SUPPLEMENTAL TEXT 3: REFERENCES**

1. Schulz J, Boklund A, Halasa THB, Toft N, Lentz HHK, Larssen KW. 2017. Network analysis of pig movements: loyalty patterns and contact chains of different holding types in Denmark. PLoS One 12:e0179915.
2. SEGES Pig Research Centre. 2015. Annual Report. <http://www.pigresearchcentre.dk/~/media/Files/PDF%20-%20Aarsberetning%20VSP%20English/VSPårsberetning%20UK%202015.pdf>.
3. Danish Agriculture and Food Council. 2015. Statistics 2014. <https://lf.dk/~/media/lf/tal-og-analyser/aarsstatistikker/statistik-svin/2014/22109-079-15-a5-lf-statistics-pigmeat-2014-uk-v4-web.pdf?la=da>.
4. European Food Safety Authority. 2009. Analysis of the baseline survey on the prevalence of methicillin-resistant Staphylococcus aureus (MRSA) in holdings with breeding pigs, in the EU, 2008 - Part A: MRSA prevalence estimates. EFSA Journal 7:1376-1458.
5. Danish Integrated Antimicrobial Resistance Monitoring and Research Programme. 2011. DANMAP 2010 - Use of antimicrobial agents and occurrence of antimicrobial resistance in bacteria from food animals, food and humans in Denmark. Statens Serum Institut, Danish Medicines Agency, National Veterinary Institute and National Food Institute, Technical University of Denmark. <http://danmap.org/~/media/Projekt%20sites/Danmap/DANMAP%20reports/Danmap_2010.ashx>.
6. Danish Integrated Antimicrobial Resistance Monitoring and Research Programme. 2015. DANMAP 2014 - Use of antimicrobial agents and occurrence of antimicrobial resistance in bacteria from food animals, food and humans in Denmark. Statens Serum Institut, National Veterinary Institute and National Food Institute, Technical University of Denmark. <http://danmap.org/~/media/Projekt%20sites/Danmap/DANMAP%20reports/Danmap_2010.ashx>.
7. European Commission. 2008. Commission decision of 20 December 2007 concerning a financial contribution from the Community towards a survey on the prevalence of *Salmonella* spp. and methicillin-resistant *Staphylococcus aureus* in herds of breeding pigs to be carried out in the Member States. <https://eur-lex.europa.eu/legal-content/EN/TXT/PDF/?uri=CELEX:32008D0055&from=EN>.
