# Supplementary material for: Drivers and Dynamics of Methicillin-Resistant Livestock-Associated Staphylococcus aureus CC398 in Pigs and Humans in Denmark
Source: mBio. 2018 Nov 13;9(6):e02142-18. doi: 10.1128/mBio.02142-18 (PMC6234867; doi:10.1128/mBio.02142-18)
Supplement: FIG S1 [file mbo005184157sf1.pdf]

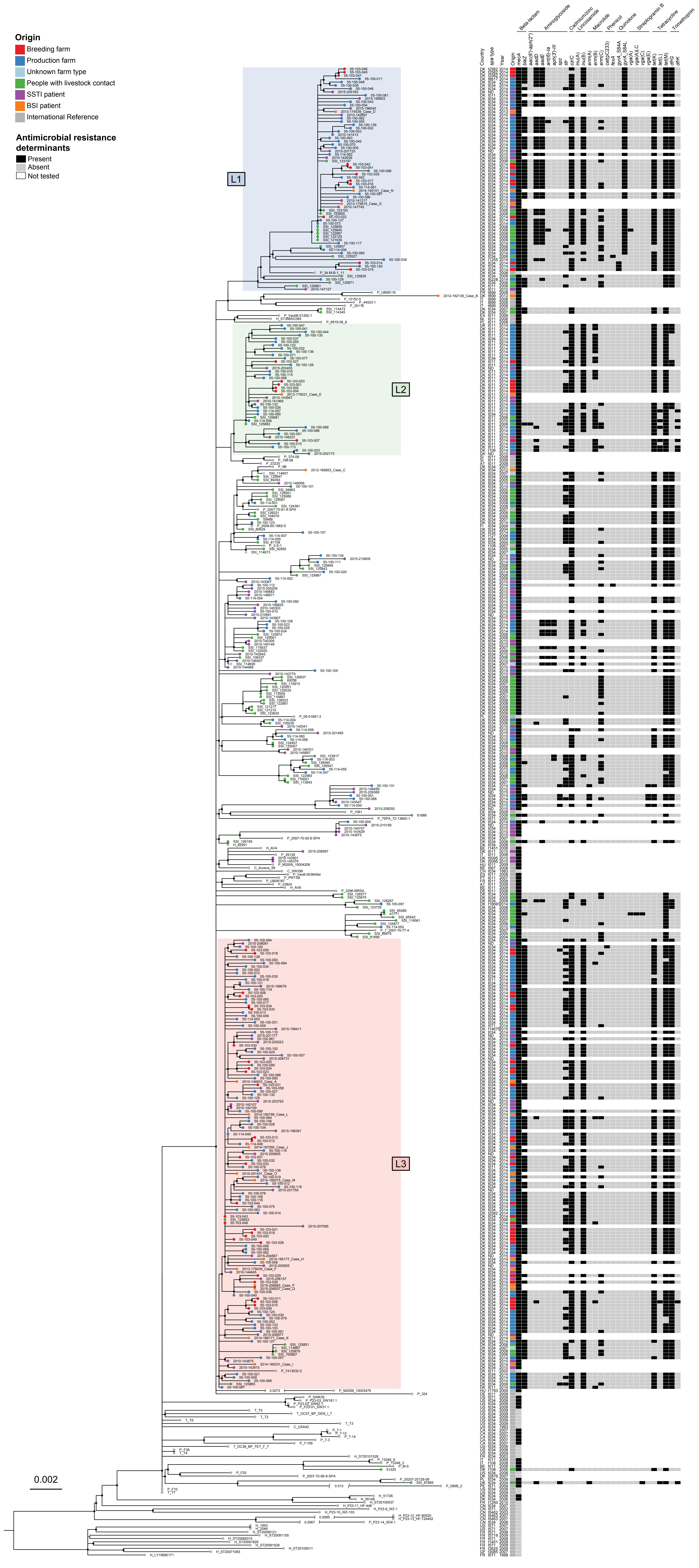

Supplemental Figure 1. Maximum-likelihood phylogeny of 288 LA-MRSA CC398 isolates from this study, 82 *S. aureus* CC398 isolates from the international reference collection, and 83 LA-MRSA CC398 isolates from Danish SSTI and BSI patients. The phylogeny was estimated for 5,529 variable sites after filtering for recombination tracts (556 SNPs), by using a GTR model of nucleotide substitution. Numbers in broken branches indicate the length by which the corresponding branch was reduced. The tree was rooted according to Price et al. (13). Bootstrap values above 90% are illustrated by filled circles at the nodes. The scale bar represents the number of nucleotide substitutions per variable site.

Abbreviations: LA-MRSA, livestock-associated methicillin-resistant *Staphylococcus aureus*; CC, clonal complex; SSTI, skin and soft tissue infection; BSI, bloodstream infection; SNP, single-nucleotide polymorphism; L1, lineage 1; L2, lineage 2; L3, lineage 3; AT, Austria; BE, Belgium; CA, Canada; CH, Switzerland; CN, China; DE, Germany; DK, Denmark; ES, Spain; FI, Finland; FR, France; GF, French Guiana; HU, Hungary; IT, Italy; NL, the Netherlands; PE, Peru; PL, Poland; PT, Portugal; SI, Slovenia; US, United States.
