# Supplementary material for: Drivers and Dynamics of Methicillin-Resistant Livestock-Associated Staphylococcus aureus CC398 in Pigs and Humans in Denmark
Source: mBio. 2018 Nov 13;9(6):e02142-18. doi: 10.1128/mBio.02142-18 (PMC6234867; doi:10.1128/mBio.02142-18)
Supplement: TABLE S2 [file mbo005184157st2.docx]

**Supplemental Table 2**. List of pig farms that participated in ≥1 survey.

| Farm type | Survey (year) | | |
| --- | --- | --- | --- |
|  | 2008 | 2010 | 2014 |
| P | - | NT | L3 |
| P | NT | - | L2 |
| P | - | NT | L1 |
| P | NT | - | L3 |
| P | - | NT | R |
| B | - | NT | L2 |
| B | - | NT | L3 |
| B | - | NT | L3 |
| B | - | NT | L1 |
| B | - | NT | L1 |
| B | - | NT | L3 |
| B | - | - | L3 |
| B | - | NT | L3 |
| B | - | NT | L2 |
| B | - | NT | L3 |
| B | - | NT | L3 |
| B | - | NT | L3 |
| B | - | NT | L1 |
| B | - | NT | L3 |
| P | - | - | NT |
| P | - | - | NT |
| P | - | - | NT |
| P | - | - | NT |
| P | - | - | NT |
| B | - | NT | - |
| B | - | NT | - |
| B | - | NT | - |
| B | - | NT | - |
| B | - | NT | - |
| P | - | NT | - |
| P | NT | - | - |

# Abbreviations: P, production farm; B, breeding farm; L1, lineage 1; L2, lineage 2; L3, lineage 3; R, remainder; -, negative; NT, not tested.
