# Supplementary material for: Drivers and Dynamics of Methicillin-Resistant Livestock-Associated Staphylococcus aureus CC398 in Pigs and Humans in Denmark
Source: mBio. 2018 Nov 13;9(6):e02142-18. doi: 10.1128/mBio.02142-18 (PMC6234867; doi:10.1128/mBio.02142-18)
Supplement: FIG S2 [file mbo005184157sf2.pdf]

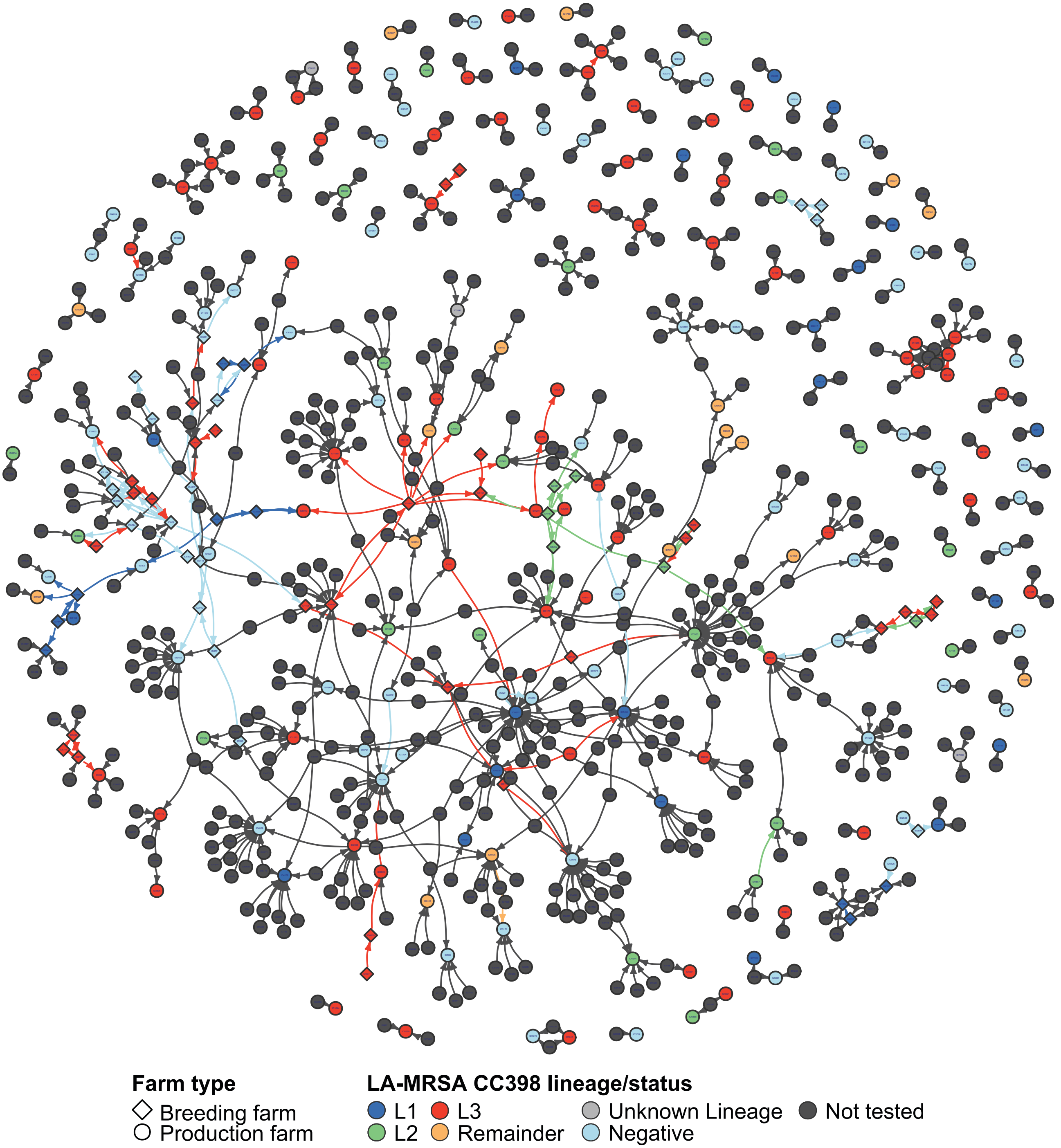

Supplemental Figure 2: Animal movements into pig farms from the 2014 survey. The network represents 17,009 pig movements into 273 farms with known LA-MRSA CC398 status, including 190 of the 207 production farms and 53 of the 66 breeding farms, over a 4-year period from 2011 to 2014. Arrows indicate the direction of pig movements. Abbreviations: LA-MRSA, livestock-associated methicillin-resistant *Staphylococcus aureus*; CC, clonal complex; L1, lineage 1; L2, lineage 2; L3, lineage 3.
