# Supplementary material for: Drivers and Dynamics of Methicillin-Resistant Livestock-Associated Staphylococcus aureus CC398 in Pigs and Humans in Denmark
Source: mBio. 2018 Nov 13;9(6):e02142-18. doi: 10.1128/mBio.02142-18 (PMC6234867; doi:10.1128/mBio.02142-18)
Supplement: TABLE S3 [file mbo005184157st3.docx]

# Supplemental Table 3. Prevalence of antimicrobial resistance determinants in the dominant lineages (L1-L3) and remainder

| Antimicrobial resistance phenotypes and determinants | No. (%) of isolates^a^ | | | | | *P* value (L1-L3 vs. R)^b^ |
| --- | --- | --- | --- | --- | --- | --- |
|  | L1  (n = 54) | L2  (n = 34) | L3  (n = 105) | L1-L3  (n = 193) | R  (n = 95) |  |
| Aminoglycoside | 47 (87)^***^ | 16 (47) | 57 (54) | 120 (62) | 46 (48) | 0.0312 |
| *aac(6')-aph(2'')* | 0 (0) | 1 (3) | 0 (0) | 1 (1) | 0 (0) |  |
| *aadD* | 41 (76)^***^ | 4 (12) | 2 (2) | 47 (24) | 3 (3) | < 0.0001 |
| *aadE* | 42 (78)^***^ | 0 (0) | 0 (0)^***^ | 42 (22) | 10 (11) | 0.0222 |
| *ant(6)-Ia* | 2 (4) | 0 (0) | 0 (0)^**^ | 2 (1) | 7 (7) | 0.0069 |
| *aph(3')-III* | 0 (0)^*^ | 0 (0) | 0 (0)^***^ | 0 (0) | 9 (9) | < 0.0001 |
| *spc* | 0 (0) | 1 (3) | 2 (2) | 3 (2) | 5 (5) |  |
| *str* | 7 (13)^**^ | 14 (41) | 55 (52)^*^ | 76 (39) | 34 (36) |  |
| β-lactam | 54 (100) | 34 (100) | 105 (100) | 193 (100) | 95 (100) |  |
| *blaZ* | 52 (96)^***^ | 1 (3) | 105 (100)^***^ | 158 (82) | 12 (13) | < 0.0001 |
| *mecA* | 54 (100) | 34 (100) | 105 (100) | 193 (100) | 95 (100) |  |
| Cadmium/zinc | 46 (85) | 34 (100)^***^ | 101 (96)^***^ | 181 (94) | 68 (72) | < 0.0001 |
| *czrC* | 46 (85) | 34 (100)^***^ | 101 (96)^***^ | 181 (94) | 68 (72) | < 0.0001 |
| Lincosamide | 43 (80)^***^ | 28 (82)^***^ | 105 (100)^***^ | 176 (91) | 15 (16) | < 0.0001 |
| *lnu*(A) | 0 (0) | 0 (0) | 0 (0) | 0 (0) | 1 (1) |  |
| *lnu*(B) | 43 (80)^***^ | 28 (82)^***^ | 105 (100)^***^ | 176 (91) | 14 (15) | < 0.0001 |
| Macrolide | 22 (41) | 24 (71)^*^ | 21 (20)^***^ | 67 (35) | 47 (49) | 0.0209 |
| *erm*(A) | 0 (0) | 0 (0) | 0 (0)^*^ | 0 (0) | 5 (5) | 0.0036 |
| *erm*(B) | 0 (0) | 23 (68)^***^ | 5 (5) | 28 (15) | 1 (1) | 0.0001 |
| *erm*(C) | 22 (41) | 4 (12)^***^ | 17 (16)^***^ | 43 (22) | 42 (44) | 0.0002 |
| Phenicol | 1 (2) | 0 (0) | 1 (1) | 2 (1) | 1 (1) |  |
| *cat*(pC233) | 0 (0) | 0 (0) | 1 (1) | 1 (1) | 0 (0) |  |
| *fexA* | 1 (2) | 0 (0) | 0 (0) | 1 (1) | 1 (1) |  |
| Quinolone | 48 (89)^***^ | 0 (0) | 0 (0) | 48 (25) | 0 (0) | < 0.0001 |
| *gyrA*_S84A | 3 (6)^*^ | 0 (0) | 0 (0) | 3 (2) | 0 (0) |  |
| *gyrA*_S84L | 45 (83)^***^ | 0 (0) | 0 (0) | 45 (23) | 0 (0) | < 0.0001 |
| Streptogramin B | 1 (2) | 0 (0) | 0 (0)* | 1 (1) | 6 (6) | 0.0059 |
| *vga*(A) | 1 (2) | 0 (0) | 0 (0) | 1 (1) | 1 (1) |  |
| *vga*(A)LC | 0 (0) | 0 (0) | 0 (0) | 0 (0) | 1 (1) |  |
| *vga*(C) | 0 (0) | 0 (0) | 0 (0) | 0 (0) | 2 (2) |  |
| *vga*(E) | 0 (0) | 0 (0) | 0 (0)* | 0 (0) | 4 (4) | 0.0113 |
| Tetracycline | 54 (100) | 34 (100) | 105 (100) | 193 (100) | 94 (99) |  |
| *tet*(K) | 48 (89)^***^ | 34 (100)^***^ | 100 (95)^***^ | 182 (94) | 57 (60) | < 0.0001 |
| *tet*(L) | 0 (0) | 6 (18)^**^ | 1 (1) | 7 (4) | 1 (1) |  |
| *tet*(M) | 54 (100) | 34 (100) | 102 (97) | 190 (98) | 94 (99) |  |
| Trimethoprim | 54 (100)^**^ | 13 (38)^***^ | 105 (100)^***^ | 172 (89) | 81 (85) |  |
| *dfrG* | 54 (100)^**^ | 7 (21)^***^ | 105 (100)^***^ | 166 (86) | 80 (84) |  |
| *dfrK* | 0 (0) | 6 (18)^**^ | 1 (1) | 7 (4) | 1 (1) |  |

Abbreviations: L1, lineage 1; L2, lineage 2; L3, lineage 3; R, remainder; *, *P* ≤ 0.1; **, *P* ≤ 0.01; ***, *P* ≤ 0.001.

^a^ Asterisks indicate significant differences between the prevalence of antimicrobial determinants between each lineage and remainder.

^b^ Only *P* values < 0.05 are shown.
